# A new type of homodiploid fish derived from the interspecific hybridization of female common carp × male blunt snout bream

**DOI:** 10.1038/s41598-017-04582-z

**Published:** 2017-06-23

**Authors:** Shi Wang, Xiaolan Ye, Yude Wang, Yuting Chen, Bowen Lin, Zhenfeng Yi, Zhuangwen Mao, Fangzhou Hu, Rurong Zhao, Juan Wang, Rong Zhou, Li Ren, Zhanzhou Yao, Min Tao, Chun Zhang, Jun Xiao, Qinbo Qin, Shaojun Liu

**Affiliations:** 1 State Key Laboratory of Developmental Biology of Freshwater Fish, Hunan Normal University, Changsha, 410081 Hunan P.R. China; 2 College of Life Sciences, Hunan Normal University, Changsha, 410081 Hunan P.R. China

## Abstract

It is commonly believed that hybridization might lead to the formation of new polyploidy species, but it is unclear whether hybridization can produce a new homodiploid species. Here, we report the spontaneous occurrence of a new crucian carp-like homodiploid fish (2n = 100) that originated from the interspecific hybridization of female common carp (*Cyprinus carpio*, *Cyprininae*, 2n = 100) × male blunt snout bream (*Megalobrama amblycephala*, *Cultrinae*, 2n = 48). The phenotype and reproductive traits of this new crucian carp-like homodiploid fish were found to be very similar to those of the existing diploid species (diploid crucian carp; *Carassius auratus*). FISH and 5S rDNA analyses revealed that the genotype of the crucian carp-like homodiploid fish differs from those of its parents but is closely related to that of diploid crucian carp. The results provide evidence of the existence of a possible route through which the distant hybridization of this cross can generate crucian carp. The new type of homodiploid fish is of great value in fish genetic breeding and for studying the early evolutionary process.

## Introduction

More than 32,500 fish species are found in nature^1^, and this number is higher than the total number of remaining vertebrate species, but the reason for this high species number is unclear. Hybridization plays a principal role in the origin of new species and appears to facilitate speciation and adaptive radiation in both animals and plants^2^. In addition, hybridization, particularly distant hybridization, should be related to the occurrence of diversified fishes. Some studies have provided important direct evidence supporting the finding that distant hybridization can lead to the formation of allotetraploid and autotetraploid fish as well as allodiploid fish^3–6^. The females and males of these new diploid and tetraploid populations are fertile, and their phenotype can be stably inherited from one generation to another. However, direct evidence showing that distant hybridization can lead to a new homoploid linage is lacking.

Homoploid hybrid speciation (HHS) is an important mode of speciation derived from interspecific hybridization that does not alter the chromosome number^7, 8^. A homodiploid lineage has two sets of chromosomes from the maternal parent into which some DNA fragments from the paternal parent are recombined. This lineage differs from an allodiploid lineage, which has two sets of chromosomes, one each from the maternal and paternal parents. Homodiploid hybrid species have been reported in several plants, e.g., *Helianthus* ^9–11^, *Vigna* ^12^, *Iris* ^13^, and *Pinus* ^14^. For example, *Helianthus anomalus* (2n = 34), a wild sunflower species, was derived from interspecific hybridization between *H. annuus* (2n = 34, ♀) and *H. petiolaris* (2n = 34, ♂)^9–11^. Molecular phylogenetic analyses have indicated that homodiploid *Helianthus* hybrids were derived independently from the parental species and are found in the most extreme *Helianthus* species habitats^11^. Rieseberg *et al*.^9^ found extensive genomic reorganization and karyotypic evolution in this homoploid hybrid, indicating the occurrence of rapid karyotypic evolution. The parental genomic structure influences genomic composition of the hybrid^9–11^. Another homodiploid plant hybrid (2n = 22) was derived from the interspecific hybridization between *Vigna umbellata* (2n = 22, ♀) and *Vigna exilis* (2n = 22, ♂), and this homodiploid hybrid is morphologically similar to *V. umbellate* and shows vigorous growth but inhabits a limestone rock mountain typically dominated by *V. exilis*, which is tolerant to drought and presents early flowering. Phylogenetic trees based on nuclear genotypes show that the homodiploid hybrid forms an independent cluster between *V. umbellata* and *V. exilis* ^12^. Based on the available evidence, researchers have proposed that homoploid hybrids can acquire novel combinations of traits and transgressive phenotypes that allow them to colonize ecological niches that are inaccessible to both parental species^13^. The above-described two cases indicate that hybridization in plants is an important evolutionary force that creates new species for adaptive evolution.

**Figure 1:**
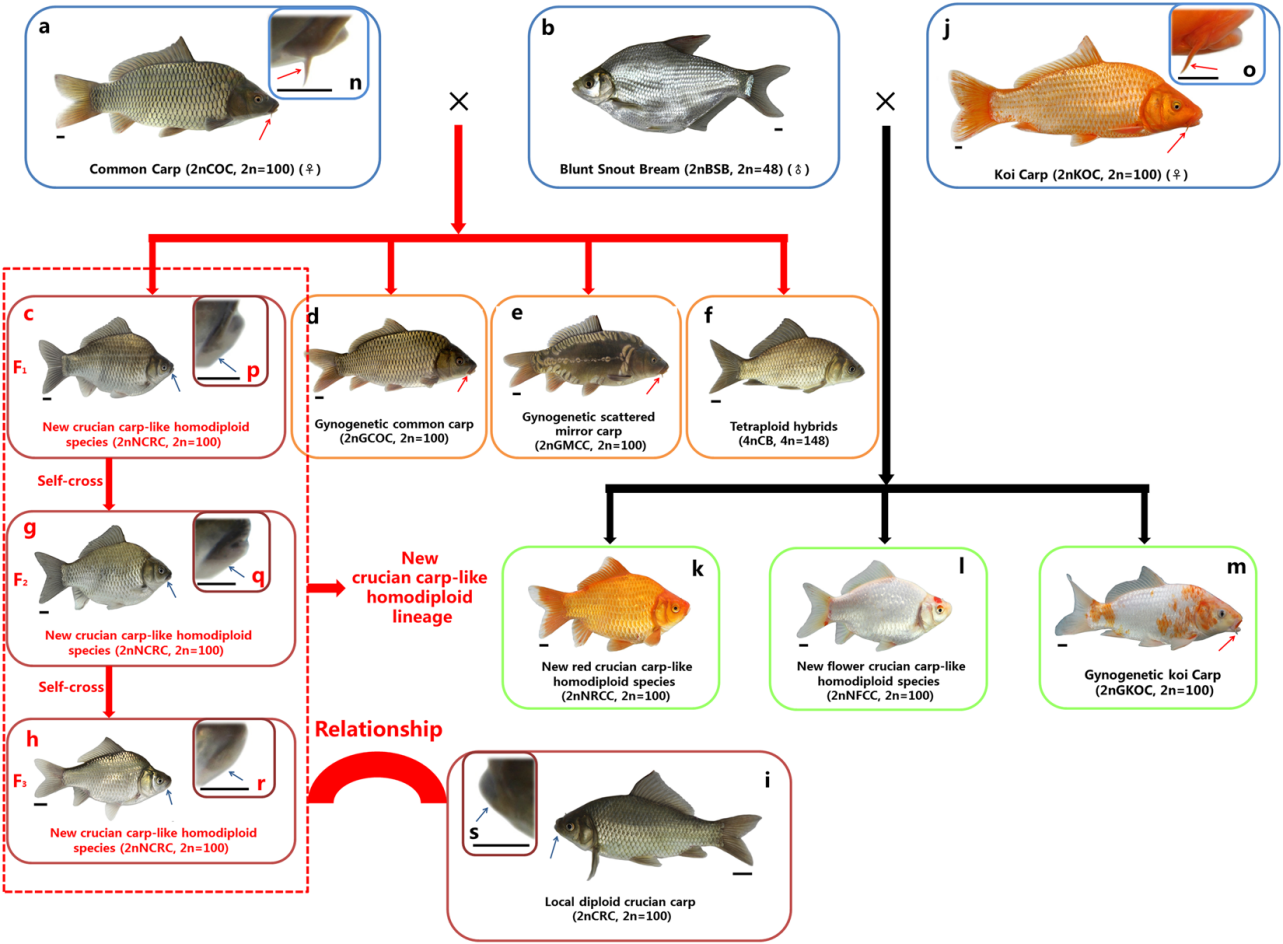
Crossing procedure, appearance of 2nCOC, 2nBSB and their offspring, appearance of 2nKOC, 2nBSB and their offspring, and appearance of local diploid crucian carp. (**a**) 2nCOC. (**b**) 2nBSB. (**c**) 2nNCRC offspring of 2nCOC (♀) × 2nBSB (♂). (**d**) 2nGCOC offspring of 2nCOC (♀) × 2nBSB (♂). (**e**) 2nGMCC offspring of 2nCOC (♀) × 2nBSB (♂). (**f**) 4nCB offspring of 2nCOC (♀) × 2nBSB (♂). (**g**) Self-cross offspring of 2nNCRC (2nNCRC-F_2_). (**h**) Self-cross offspring of 2nNCRC-F_2_ (2nNCRC-F_3_). (**i**) Local diploid crucian carp (2nCRC). (**j**) Koi carp (2nKOC). (**k**) Red crucian carp-like homodiploid fish (2nNRCC). (**l**) Flower crucian carp-like homodiploid fish (2nNFCC). (**m**) Gynogenetic koi carp (2nGKOC). (**n**) Amplification of the head of 2nCOC (**a**). (**o**) Amplification of the head of 2nKOC (**j**). (**p**) Amplification of the head of 2nNCRC (**c**). (**q**) Amplification of the head of 2nNCRC-F_2_ (**g**). (**r**) Amplification of the head of 2nNCRC-F_3_ (**h**). (**s**) Amplification of the head of 2nCRC (**i**). The red arrows indicate barbels, and the blue arrows indicate barbel loss. Scale bars in a–s, 1 cm.

HHS is very rare in fish. This manuscript reports a crucian carp-like homodiploid fish derived from the interspecific hybridization of female common carp × male blunt snout bream, and the results provide insights into the role of interspecific hybridization in homoploid speciation in fish. The common carp (*Cyprinus carpio*, 2n = 100, abbreviated 2nCOC; Fig. 1a) and crucian carp (*Carassius auratus*, 2n = 100, abbreviated 2nCRC; Fig. 1i) are very important, widely cultured fish species in Eurasia and America. These two species belong to the same family (*Cyprinidae*) and subfamily (*Cyprininae*) but different genera (*Cyprinus* and *Carassius*, respectively). In terms of their taxonomic status, 2nCOC and 2nCRC are considered close even though they are classified into different genera, but the reasons underlying their close and evolutionary relationships are unclear.

More than one hundred years ago, Darwin^15^ described “strongly marked varieties or doubtful species” when considering the boundary between what we now consider conspecific populations (or subspecies) and sibling species. The growing popularity of whole-genome sequencing has resulted in the genome sequencing of an increasing number of species, and analyses of these genomes will provide more evidence of gene flow, which scientists predict is very common between species. De Manuel *et al*. showed that this type of “reticulate” evolution, which involves divergence with continuous genetic exchange, is shared among nonhominid great apes and among hominids^16^. Recent research has shown that chimpanzees and bonobos show signs of ancestral gene exchange and thus provide insight into the dynamics of speciation^17^.

Through artificial hybridization, we previously showed that the interspecific hybridization of female red crucian carp (no barbels) × male blunt snout bream (no barbels) forms tetraploid hybrids that contain a pair of barbels. This tetraploid hybrid shows a portion of the original genes of common carp^18–20^. Based on their taxonomic status, common carp and crucian carp share a close relationship even though they are classified into different genera, but their evolutionary relationship remains unclear. In this study, we designed an interspecific hybridization of female common carp (two pairs of barbels) × male blunt snout bream (no barbels) to form a new crucian carp-like homodiploid fish without barbels. The genome of the crucian carp-like homodiploid fish contains that of the common carp, and the results indicate the existence of gene flow between common carp and crucian carp under natural conditions. Diploid crucian carp might originate from the cross of female common carp × male blunt snout bream. However, low levels of ongoing mixing cannot be easily distinguished from discreet periods of gene flow, but the genomic variation and morphological changes (e.g., changes in barbels) suggest wind-driven gene flow.

To study the genetic variation in the hybrid genome, we investigated the 5S rDNA variation. In eukaryotes, the 5S rDNA multigene family occurs as several thousand copies of tandem repeated units, comprising a highly conserved coding sequence of 120 bp and nontranscribed flanking DNA (nontranscribed spacer; NTS) containing some elements regulating the transcription of the coding sequence^21–23^, which are typically organized in a tandem array(s) in one or more chromosome loci^24^. Although the *5S rRNA* gene is highly conserved, even between unrelated species, the NTSs show variations in both length and sequence. These variations are due to insertions/deletions, mini-repeats, and base substitutions and have been used as species-specific or population-specific markers in evolutionary studies^25, 26^. These mechanisms maintain a high sequence similarity between copies and prevent the independent evolution of each member of a multi-gene family^27^. The NTS regions appear to be subject to rapid evolution, which makes them important for studies concerning the organization and evolution of the *5S* multi-gene family and as markers for tracing recent evolution events^28, 29^. Although a number of studies have examined the structural and functional organization of the 5S rDNA arrays in bony fishes^22, 23, 30–32^, relatively few studies have performed similar investigations with hybrids^33–35^.

## Results

### Formation of experimental fish

The diploid common carp (*Cyprinus carpio*, 2n = 100, abbreviated 2nCOC; Fig. 1a) is widely cultured in Eurasia and America and belongs to the family *Cyprinidae* and subfamily *Cyprininae*. The diploid blunt snout bream (*Megalobrama amblycephala*, 2n = 48, abbreviated 2nBSB; Fig. 1b), which is cultured in Asia, belongs to the family *Cyprinidae* and subfamily *Cultrinae*. During the reproductive season (from April to June) in 2014–2016, distant hybridization of 2nCOC (♀) × 2nBSB (♂) repeatedly produced four ploidy types (Fig. 1 and Table 1, and Supplementary Fig. S1), including the new crucian carp-like homodiploid fish (2n = 100, 2nNCRC; 1.50–1.90%; Fig. 1c), a diploid gynogenesis common carp (2n = 100, 2nGCOC; 75.80–76.90%; Fig. 1d), a diploid gynogenesis scattered mirror carp (2n = 100, 2nGMCC; 16.50–18.20%; Fig. 1e), and a tetraploid hybrid (4n = 148, 4nCB; 4.00–4.80%; Fig. 1f). However, reverse crosses of 2nBSB (♀) × 2nCOC (♂) did not yield any surviving progeny. Interestingly, the morphology of the new crucian carp-like homodiploid fish (2nNCRC) is very similar to that of the local crucian carp (*Carassius auratus*, 2n = 100, abbreviated 2nCRC; Fig. 1c,g, and h vs. 1i), suggesting that interspecific hybridization between two different species can lead to HHS. 2nCOC and 2nCRC belong to the same family (*Cyprinidae*) and subfamily (*Cyprininae*) but different genera (*Cyprinus* and *Carassius*, respectively). One varying feature of 2nCOC is scattered scales on the body, resulting in its name of scattered mirror carp (2n = 100, abbreviated 2nMCC). There are two varieties of 2nCRC, specifically red crucian carp (2n = 100, abbreviated 2nRCC) and Japanese crucian carp (2n = 100, abbreviated 2nJCC), which are characterized by red and white scales on the body, respectively.

**Table 1:** Percentage of different types of offspring of 2nCOC (♀) x 2nBSB (♂).

Year	Fish type			
	2nNCRC	2nGCOC	2nGMCC	4nCB
2014	1.50%	75.80%	18.20%	4.50%
2015	1.90%	76.30%	17.80%	4.00%
2016	1.80%	76.90%	16.50%	4.80%

Our laboratory also performed other interesting hybridization experiments that showed that the interspecific hybridization of female koi carp (*Cyprinus carpio haematopterus*, 2n = 100, 2nKOC; Fig. 1j) × male blunt snout bream repeatedly produced three types, including red crucian carp-like homodiploid fish without barbels (2n = 100, 2nNRCC; Fig. 1k), flower crucian carp-like homodiploid fish without barbels (2n = 100, 2nNFCC; Fig. 1l), and diploid gynogenesis koi carp (2n = 100, 2nGKOC; Fig. 1m) with two pairs of barbels. The koi carp and common carp belong to the same genera (*Cyprinus*) but different varieties. The interspecific hybridization of female koi carp × male blunt snout bream repeatedly produced two types of new crucian carp-like homodiploid fish and one type of diploid gynogenesis carp. Interestingly, the morphologies of the two types of new crucian carp-like homodiploid fish (2nNRCC and 2nNFCC) are very similar to that of 2nRCC, suggesting that interspecific hybridization between two different species can lead to HHS. By contrast, the interspecific hybridization of female red crucian carp × male blunt snout bream forms tetraploid hybrids with a pair of barbels. These tetraploid hybrids show a portion of the original genes of common carp^18–20^. The interesting hybridization experiments suggest the potential existence of gene flow between *Cyprinus carpio* and *Carassius auratus* under natural conditions. Diploid crucian carp might originate from the cross of female common carp × male blunt snout bream.

Furthermore, 2nNCRC males and females mated to produce the second diploid generation (2n = 100, 2nNCRC-F_2_; Fig. 1g and Supplementary Fig. S1), but only 3.83% of the 2nNCRC (F_1_) individuals survived embryogenesis. By the F_3_ generation of the self-cross, the survival rate of 2nNCRC increased to 82.67% (Table 2). To that end, the new bisexual fertile crucian carp-like homodiploid fish lineage derived from distant hybridization was established to provide a good model system for the study of evolutionary genetics. Furthermore, given its attractive appearance and rapid growth rate (2nNCRC can grow to more than 500 g a year, whereas other diploid crucian carp can only grow to approximately 350 g a year, indicating that 2nNCRC shows obvious heterosis), the new crucian carp-like homodiploid fish has potential economic value for aquaculture.

**Table 2:** Average survival ratios of early generations of 2nNCRC embryos from 2nCOC (♀) x 2nBSB (♂).

2nNCRC	No. of fertilized eggs	No. of embryos in hatching period			Hatching rate (%)	P value (t-test)
		Total	Mean	SD		
F_1_	6 × 100	23	3.83	1.46	3.83 ± 1.46	<0.01
F_2_	6 × 100	485	80.83	1.16	80.83 ± 1.16	<0.01
F_3_	6 × 100	496	82.67	1.08	82.67 ± 1.08	<0.01

SD: Standard deviation. Hatching rate = number of surviving embryos at the stage of hatching/number of fertilized eggs × 100%. Embryonic development was observed at a water temperature of 22 °C using a Meiji RZ Stereo microscope. One hundred fertilized eggs selected at random from each group were examined, and six replicates of each fish were included. The criteria for evaluating the early embryonic development of 2nNCRC were described by Liu (2001).

### Measurement of DNA content, examination of chromosome number and formation of karyotype

We used the sum of the DNA contents of 2nCOC and 2nBSB as the controls. The distribution of the DNA content among all of the hybrid offspring is illustrated in Supplementary Table S1 and Supplementary Fig. S2. The mean DNA contents of the 2nNCRC and 2nNCRC-F_2_ hybrids were equal to that of 2nCOC (*P* > 0.05), suggesting that 2nNCRC and 2nNCRC-F_2_ have two sets of 2nCOC-derived chromosomes (Supplementary Table S1 and Supplementary Fig. S2). The value of the DNA content was directly proportional to the degree of chromosomal ploidy, i.e., a high chromosomal ploidy was associated with increased DNA content. Ploidy analyses of the chromosomal number and karyotype (Supplementary Table S2 and Supplementary Fig. S3) revealed that 2nNCRC and 2nNCRC-F_2_ have 100 chromosomes with karyotypes (22 m + 34sm + 22st + 22t) consistent with those of 2nCOC and 2nCRC, 2nRCC, or 2nJCC. The chromosomal spreads were examined to directly identify the chromosomal number, and the results suggest that 2nNCRC is diploid and has two sets of chromosomes derived from 2nCOC.

### Fluorescence *in situ* hybridization

**Figure 2:**
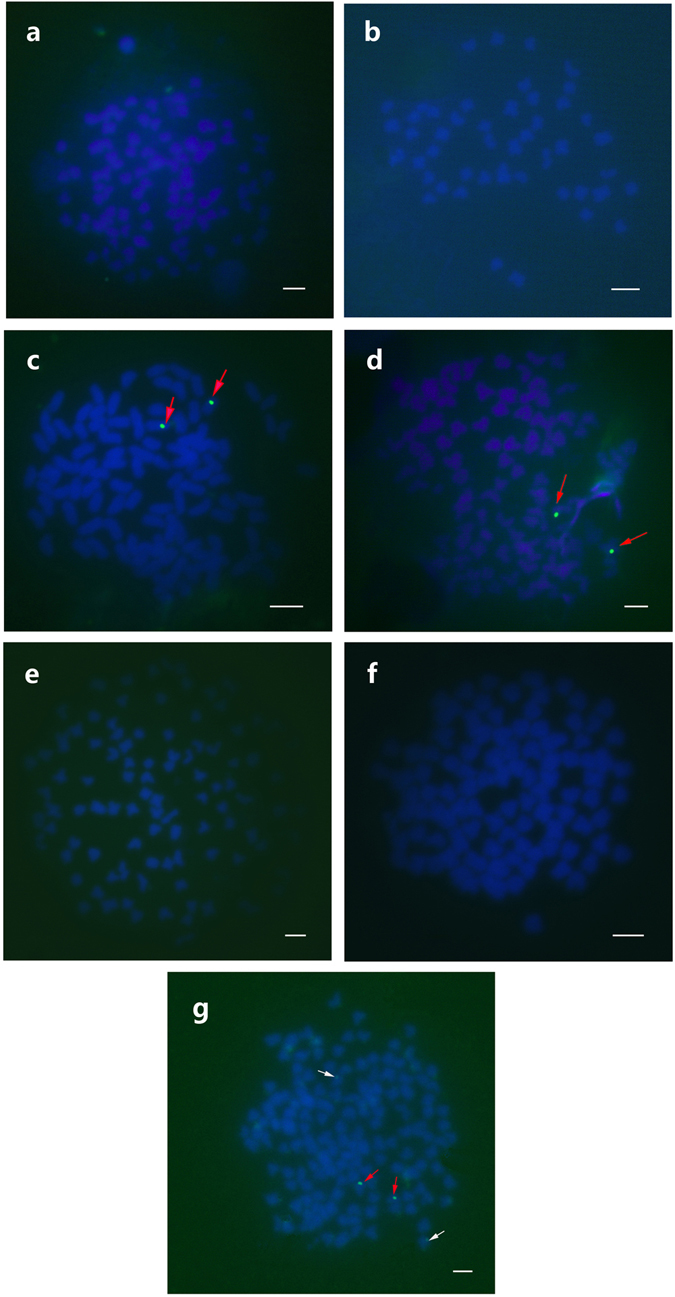
Examination of FISH signals in 2nCOC, 2nBSB, 2nNCRC, 2nNCRC-F_2_, 2nGCOC, 2nGMCC and 4nCB. (**a**) 2nCOC does not have a *5S* gene locus. (**b**) 2nBSB does not have a *5S* gene locus. (**c**) 2nNCRC has two strong fluorescence signals (red arrows). (**d**) 2nNCRC-F_2_ has two strong fluorescence signals (red arrows). (**e**) 2nGCOC does not have a *5S* gene locus. (**f**) 2nGMCC does not have a *5S* gene locus. (**g**) 4nCB has two strong (red arrows) and two weak (white arrows) fluorescence signals. Scale bars in a–g, 3 μm.

A fluorescence *in situ* hybridization (FISH) analysis using 5S rDNA as a probe (GenBank Accession No. GQ485556) revealed two strong fluorescence signals in 2nNCRC and 2nNCRC-F_2_, two strong and two weak fluorescence signals in 2nCRC, 2nRCC, and 4nCB, and no signal in 2nCOC, 2nBSB, 2nGCOC, or 2nGMCC (Fig. 2a–g). The FISH analysis also identified the heredity and variation of the chromosomes in the hybrids at the molecular level, and the FISH results suggest that 2nNCRC (F_1_–F_3_) is very close to 2nCRC.

### Morphological traits

The main morphological differences between 2nCOC and 2nCRC, 2nRCC, or 2nJCC are that 2nCOC has two pairs of barbels and 35–38 lateral scales, whereas the other types of crucian carp have no barbels and 27–34 lateral scales. In addition, 2nBSB has no barbels and 49–52 lateral scales (Tables 3 and 4 and Fig. 1). Significant differences (*P* < 0.01) in most of the qualitative and quantitative traits were found between 2nNCRC and 2nCOC (Tables 3 and 4 and Fig. 1), with the most obvious being that 2nNCRC has no barbels, unlike its female parent. Additionally, 2nNCRC has fewer lateral scales than 2nCOC (*P* < 0.01; Table 3). However, the HL/BL ratio of 2nNCRC was significantly greater than those of 2nCOC and 2nBSB. The BW/BL and HW/BW ratios (*P* < 0.01) of 2nNCRC were between those of 2nCOC and 2nBSB. Interestingly, most of the qualitative and quantitative traits of 2nNCRC, including barbel loss and few lateral scales, were similar to those of 2nCRC (Tables 3 and 4 and Fig. 1). However, several morphological differences between 2nNCRC and 2nCRC, including the number of lateral scales, were detected: the number of lateral scales found in 2nNCRC was between that of 2nCRC and that of 2nCOC, although it was closer to that of 2nCRC. A comparison of the qualitative and quantitative traits between the hybrid progenies and their parents helped identify the similarities and differences between them. This morphological analysis indicates that 2nNCRC is more phenotypically similar to 2nCRC than its parents.

**Table 3:** Comparison of the countable traits between the different offspring and their parents.

Fish type	No. of lateral scales	No. of upper lateral scales	No. of lower lateral scales	No. of dorsal fins	No. of abdominal fins	No. of anal fins
2nCOC	36.35 ± 1.43(35–38)	5.37 ± 0.45(5~6)	5.30 ± 0.43(5~6)	III + 17.62 ± 0.89(III + 17~19)	8.58 ± 0.51(8~9)	III + 6.37 ± 0.39(III + 6~7)
2nBSB	50.60 ± 1.20(49–52)	9.48 ± 0.55(9~10)	10.19 ± 0.97(9~11)	III + 8.50 ± 0.52(III + 8~9)	9.18 ± 0.69(8~10)	III + 25.90 ± 0.88(III + 25~27)
2nGCOC	36.50 ± 0.50(36–37)	5.75 ± 0.38(5~6)	6.00 ± 1.00(5~7)	III + 18.30 ± 1.32(III + 17~20)	9.39 ± 1.27(8~11)	IIIIII + 8.52 ± 1.11(III + 7~10)
2nGMCC	**\**	**\**	**\**	III + 18.34 ± 1.32(III + 17~20)	9.00 ± 1.00(8~10)	III + 8.51 ± 1.12(III + 7~10)
2nNCRC	29.35 ± 0.38(29–30)	6.43 ± 0.52(6~7)	7.36 ± 0.56(7~8)	III + 18.34 ± 1.28(III + 17~20)	9.08 ± 1.69(7~11)	IIIIII + 7.66 ± 1.25(III + 6~9)
4nCB	30.94 ± 0.86(30–32)	6.00 ± 0.00(6)	6.45 ± 0.42(6~7)	III + 17.94 ± 1.67(III + 16~20)	8.36 ± 1.29(7~10)	III + 7.99 ± 1.67(III + 6~9)
2nRCC	29.15 ± 0.79(28–30)	5.65 ± 0.36(5–6)	6.35 ± 0.49(6~7)	III + 19.05 ± 0.85(III + 18~20)	8.39 ± 0.49(8~9)	III + 6.38 ± 0.37(III + 6~7)
2nJCC	33.14 ± 0.86(32–34)	7.24 ± 1.06(6–8)	6.03 ± 0.82(5~7)	III + 19.14 ± 0.78(III + 18~20)	9.16 ± 0.89(8~10)	III + 6.49 ± 0.41(III + 6~7)
2nCRC	28.48 ± 1.28(27–30)	5.85 ± 0.83(5~7)	6.00 ± 0.00(6)	III + 17.02 ± 0.89(III + 16~18)	8.45 ± 1.32(7~10)	III + 7.54 ± 1.57(III + 6~9)

Uppercase Roman numerals indicate the number of spines, and Arabic numerals reflect the number of soft fins. The numbers before and after the symbol “ ± ” present the mean and standard deviation of the numbers of fins, respectively, and “~” indicates the range of the measured number of fins.

**Table 4:** Comparison of the measurable traits between the offspring and their parents.

Fish type	BL/WL	BW/BL	HL/BL	HW/HL	TW/TL	HW/BW
2nCOC	0.83 ± 0.07	0.34 ± 0.01	0.24 ± 0.02	0.81 ± 0.07	0.86 ± 0.11	0.60 ± 0.01
2nBSB	0.84 ± 0.04	0.41 ± 0.04	0.20 ± 0.04	0.88 ± 0.03	0.93 ± 0.04	0.49 ± 0.04
2nGCOC	0.83 ± 0.01	0.35 ± 0.01	0.27 ± 0.02	0.81 ± 0.03	0.84 ± 0.02	0.67 ± 0.03
2nGMCC	0.83 ± 0.05	0.34 ± 0.02	0.28 ± 0.02	0.81 ± 0.06	0.87 ± 0.10	0.66 ± 0.03
2nNCRC	0.84 ± 0.02	0.41 ± 0.02	0.26 ± 0.01	0.88 ± 0.02	0.88 ± 0.03	0.56 ± 0.01
4nCB	0.83 ± 0.02	0.38 ± 0.02	0.27 ± 0.01	0.88 ± 0.02	0.89 ± 0.01	0.62 ± 0.01
2nRCC	0.81 ± 0.03	0.45 ± 0.01	0.27 ± 0.01	0.85 ± 0.04	1.27 ± 0.02	0.51 ± 0.02
2nJCC	0.81 ± 0.02	0.45 ± 0.03	0.27 ± 0.02	0.85 ± 0.06	1.23 ± 0.02	0.51 ± 0.03
2nCRC	0.81 ± 0.03	0.37 ± 0.03	0.27 ± 0.02	0.95 ± 0.10	1.08 ± 0.15	0.69 ± 0.07

### Fertility and size of gametes

**Figure 3:**
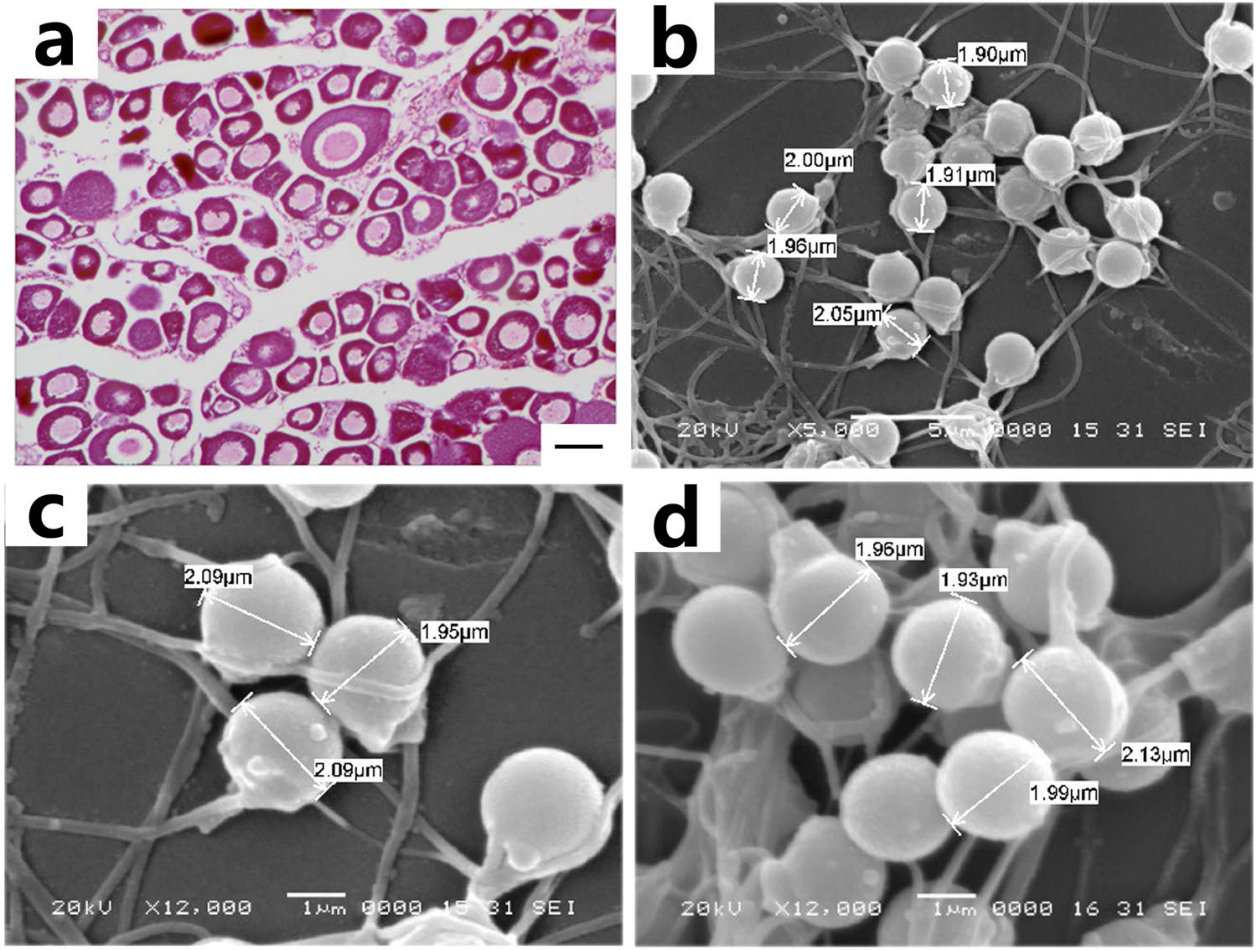
Ovarian microstructure of 2nNCRC and structure of 2nNCRC, 2nGCOC, and 2nGMCC sperm. (**a**) Ovarian microstructure of 2nNCRC; bar = 20 μm. (**b**) Structure of 2nNCRC sperm under a scanning electron microscope; bar = 5 μm. (**c**) Structure of 2nGCOC sperm under a scanning electron microscope; bar = 1 μm. (**d**) Structure of 2nGMCC sperm under a scanning electron microscope; bar = 1 μm.

Our analysis of reproductive traits revealed that 2nCOC and 2nBSB reach sexual maturity at two years of age, whereas 2nCRC and its varieties (2nRCC and 2nJCC) are sexually mature at the age of one year. 2nNCRC reaches sexual maturity at one year of age, whereas 2nGCOC, 2nGMCC, and 4nCB reach sexual maturity at two years. The ovaries of 6-month-old 2nNCRC are fully developed. Many oogonia were found to show massive proliferation for the development of phase II oocytes (Fig. 3a). Furthermore, large numbers of eggs were stripped from 1-year-old 2nNCRC females. Observations of gonadal development are important for assessing the fertility of the hybrid progenies. The white semen can be stripped out from the male 2nNCRC individuals at the age of 6 months and from the male 2nGCOC and 2nGMCC individuals at the age of 18 months. The spermatozoa of 6-month-old 2nNCRC, 18-month-old 2nGCOC, and 18-month-old 2nGMCC were compared under a scanning electron microscope, which revealed that the heads and tails of the sperm produced by these male hybrids are well-developed (Fig. 3b–d). The size of the head of the 2nNCRC sperm (Fig. 3b) was similar to that of the 2nGCOC and 2nGMCC sperm (Fig. 3c,d). The mean diameter of the 2nNCRC haploid sperm was 1.96 μm, whereas those of the 2nGCOC and 2nGMCC haploid sperm were 2.04 μm and 2.00 μm, respectively. The sizes of the mature gametes were recorded to identify the ploidy of the gametes. The 2nNCRC males and females were found to exhibit normal gonadal development (Fig. 3) and produce mature sperm and eggs, which can fuse to form 2nNCRC-F_2_. In fact, 2nNCRC-F_3_ was obtained from the mating of 2nNCRC-F_2_ males and females, which suggests that 2nNCRC can be stably inherited from one generation to the next and forms a stable lineage (F_1_–F_2_–F_3_). These results also support the conclusion that 2nNCRC is similar to 2nCRC in terms of reproductive traits (reaching sexual maturity at the age of one year).

### Genetic and variation analysis of 5S rDNA sequences

5S rDNA has traditionally been used as a genomic DNA marker for tracing evolutionary events^28, 29^, and we thus analysed the 5S rDNA in 2nCOC (♀), 2nBSB (♂), and their four offspring types. Based on the significant amount of variation obtained among *5S* coding and NTS sequences in this and other studies, sufficient numbers of clones must be sequenced to ensure that orthologous genes are used in the analysis (Supplementary Table S3). PCR amplification generated 5S rDNA units of different sizes within the same species due to size polymorphisms of the NTSs. Distinct electrophoretic patterns were obtained among the different fishes (Supplementary Fig. S4).

**Figure 4:**
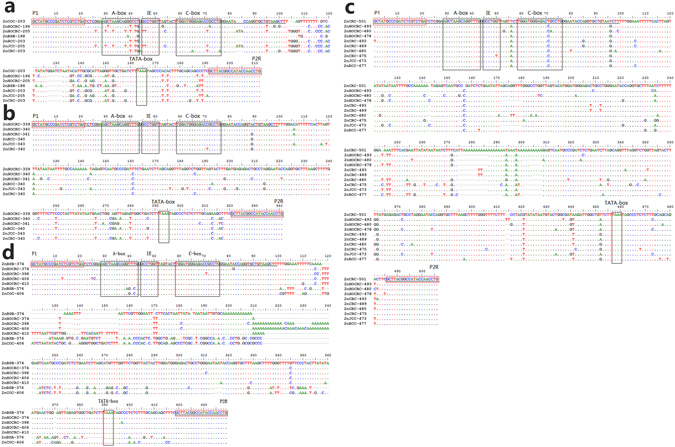
Nucleotide sequence alignment of sequenced 5S rDNA fragments in 2nNCRC, 2nCOC, 2nBSB, 2nCRC, 2nRCC, and 2nJCC. (**a**) Nucleotide sequence alignment of 5S rDNA fragments (class-I). (**b**) Nucleotide sequence alignment of 5S rDNA fragments (class-I-V_1_) between 2nNCRC, 2nCRC, 2nRCC and 2nJCC. (**c**) Nucleotide sequence alignment of 5S rDNA fragments (class-I-V_2_) between 2nNCRC, 2nCRC, 2nRCC and 2nJCC. (**d**) Nucleotide sequence alignment of 5S rDNA fragments (class-II-V_1_). The *5S rRNA gene* coding region is underlined. The regulatory sequences [A-box (AGCTAAGCAGGGTCG/AGCTAAGCAGGTTTG), intermediate element (GCCTGGT), C-box (TGGATGGGAGACCGCCTG) and TATA-box (TAAA)] are shown in black boxes. The PCR primers (P1 and P2R) are shown in red boxes. The dots indicate sequence identity, and the hyphens represent insertions/deletions.

Two 5S rDNA bands (203 and 406 bp) were detected in 2nCOC, and both of these belong to class I 5S rDNA (203 bp). The 406-bp band is likely an indicator of 203-bp class-I 5S rDNA duplication. The analysis of 2nBSB also revealed two bands (188 bp and either 376 or 374 bp) belonging to class-II (188 bp) and class-II-V_1_ (374 bp). The 376-bp band is likely an indicator of 188-bp class-II 5S rDNA duplication, and the 374-bp band is a variant of the 376-bp band. 2nNCRC showed four bands: the first band belongs to class-I-V_1_ (196 or 205 bp), the second band was classified as class-I-V_2_ (339, 340, or 341 bp), the third band belongs to class-II-V_1_ (374, 398, 406, or 410 bp), and the fourth band was found to belong to class-I-V_3_ (478, 480, or 493 bp) (Supplementary Fig. S4 and Supplementary Table S3). BLAST results revealed that the 2nNCRC 5S rDNA class-I-V_1_ is a variant of the 203-bp 5S rDNA class-I of its female parent (2nCOC; Fig. 4a). Interestingly, the 2nNCRC 196-bp 5S rDNA class-I-V_1_ exhibited high nucleotide identity (92.10%) with the 2nRCC 203-bp 5S rDNA (GenBank Accession No. GQ485555.1). However, a 7-bp deletion and nine mutations were detected (Fig. 4a), revealing substantially higher identity than that (80.50%) between the 2nNCRC 196-bp 5S rDNA class-I-V_1_ and the 2nCOC 203-bp 5S rDNA class-I. Similarly, high nucleotide identity (99.50%) was observed between the 2nNCRC 205-bp 5S rDNA class-I-V_1_ and the 2nJCC 205-bp 5S rDNA (GenBank Accession No. KR706447.1), and only one mutation was found in 2nNCRC (Fig. 4a). This identity was markedly higher than that (78.30%) between the 2nNCRC 205-bp 5S rDNA class-I-V_1_ and the 2nCOC 203-bp 5S rDNA class-I. The 340-bp 5S rDNA class-I-V_2_, which contains five mutations, as shown in the new 2nNCRC band (Fig. 4b), exhibited high nucleotide identity (98.50%) with the 2nCRC 340-bp 5S rDNA (GenBank Accession No. DQ659273.1), and this identity was found to be substantially higher than that (78.10%) between the 2nNCRC 340-bp 5S rDNA class-I-V_2_ and the 2nCOC 203-bp 5S rDNA class-I. In addition, 92.40% identity was observed between the 2nNCRC 493-bp 5S rDNA class-I-V_3_ and the 2nCRC 501-bp 5S rDNA (GenBank Accession No. GU188690.1), which presented a 12-bp deletion, three (1-bp, 1-bp and 2-bp) insertions, and 22 mutations. A lower identity of 83.74% was observed between the 2nNCRC 493-bp 5S rDNA class-I-V_3_ and the 2nCOC 203-bp 5S rDNA class-I (Fig. 4c). The complete 5S rDNA analyses provide further evidence that 2nNCRC is closely related to 2nCRC at the genomic DNA level. However, a high nucleotide identity (95.70%) was observed between the 2nNCRC 374-bp 5S rDNA class-II-V_1_ and the 2nBSB 374-bp 5S rDNA class-II-V_1_, where there is a 2-bp deletion, three (4-bp, 1-bp, and 1-bp) insertions, and four mutations. A lower identity was observed (82.27%) between the 2nNCRC 374-bp 5S rDNA class-II-V_1_ and the 2nCOC 203-bp 5S rDNA class-I (Fig. 4d). These results indicate the recombination of 2nBSB genomic DNA into the 2nNCRC genome, leading to clear changes in the 2nNCRC genome. Moreover, elimination of part of the paternal 5S rDNA unit (2nBSB 188-bp 5S rDNA class II) in 2nNCRC during hybridization gave rise to genomic shock in the offspring of the distant hybridization.

The DNA fragments derived from 2nBSB that were recombined into the 2nCOC-derived genome resulted in many variants in the 2nNCRC genome, including the presence of chimaeric genes, deletions, insertions, and mutations. These genetic variations were maintained in F_2_ through purifying selection. The altered 5S rDNA structure of 2nNCRC is very close to that of 2nCRC, providing further evidence that 2nNCRC is more closely related to 2nCRC at the genomic DNA level.

## Discussion

In plants, homoploid speciation is a rapid evolutionary route observed in nature^36, 37^. Fertile and stable hybrid neospecies have been experimentally developed in several plant genera^38^, and molecular phylogenetic evidence indicates that homodiploid plant hybrids are independently derived from their parental species, e.g., *Helianthus* and *Vigna* ^9–12^. However, homoploid speciation is very rare in fish. Homodiploid species of fish derived from distant hybridization, which does not include chromosomal duplication, typically show unequal parental genomic contributions, which might result in genomic incompatibilities and genomic shock. These effects are likely responsible for the rareness of homodiploid fish species. Incompatibilities due to differences in chromosome number between the parent species will result in genomic shock in the F_1_ offspring of distant hybridization, which might lead to their mortality. In this study, the 2nCOC (♀, 2n = 100) × 2nBSB (♂, 2n = 48) cross yielded living F_1_ offspring, whereas the reverse cross, 2nBSB (♀, 2n = 48) × 2nCOC (♂, 2n = 100), did not produce any living offspring, suggesting incompatibilities due to the difference in the chromosome number between the parents. Our previous study^39^ indicated that distant hybridization in fish could produce living offspring if the chromosome number of the maternal parent is higher than or equal to that of the paternal parent; however, if the maternal parent has less chromosomes than the paternal parent, it is very difficult to obtain living offspring. In addition, incompatibilities due to changes in DNA structure will lead to genomic shock in the F_1_ offspring, which might result in their mortality. Some homoploid plants, such as sunflowers^9^, exhibit genomic reorganization and massive gene loss. The data obtained in this study for 2nNCRC revealed that elimination of part of the paternal 5S rDNA unit (2nBSB 188-bp 5S rDNA class II) and variations in the maternal 5S rDNA unit (2nCOC 203-bp 5S rDNA class I) and chromosomal loci. The unusual genomic DNA variations observed in 2nNCRC, which exhibited very low survival rates (1.50–1.90%, Table 1), suggest the effects of genomic incompatibilities in the process of homodiploid fish speciation. The incompatibilities at the chromosome and genomic DNA levels that arise from distant hybridization result in genomic shock in the offspring, and this process might be responsible for the rarity of homodiploid speciation in vertebrates.

However, once the chromosomal and genomic DNA incompatibilities are overcome, a small number of homodiploid fish, occurring at very low frequency, can survive. As of 2014, we observed only six surviving 2nNCRC, representing a very low percentage (1.50–1.90%, Table 1) of F_1_ embryos of the 2nCOC (♀) × 2nBSB (♂) cross. These individuals successfully overcame the chromosomal and genomic DNA incompatibilities and spontaneously formed homodiploid fish. Despite the very low frequency of F_1_ 2nNCRC individuals, the F_1_ females and males were fertile and thus mated to each other to produce the next generation, which in turn was used to produce the F_3_ generation. The numbers of F_2_ and F_3_ individuals of 2nNCRC were 500 (in 2015) and 100,000 (in 2016), respectively, revealing that the numbers increased with subsequent generations. This process provides a good foundation for the formation of a stable lineage, potentially of a new homodiploid species. Our findings provide direct evidence of the formation of homodiploid species in the animal kingdom and suggest that interspecific hybridization between two different species can lead to a new homoploid species.

The 2nCOC and 2nCRC, which are very popular aquaculture species in Asia, belong to the same family (*Cyprinidae*) and subfamily (*Cyprininae*) but different genera (*Cyprinus* and *Carassius*, respectively). However, the reason for their close relationship has not been demonstrated. In theory, these two species are related in terms of their evolutionary route, but prior to this study, direct evidence proving their evolutionary relationship was lacking. However, the results obtained in the present study provide evidence indicating the potential route through which the distant hybridization of 2nCOC (♀) × 2nBSB (♂) can generate crucian carp. Most of the qualitative and quantitative morphological traits of 2nNCRC, including barbel loss and few lateral scales, were found to be more closely related to those of 2nCRC (Tables 3 and 4 and Fig. 1) and are significantly different from those of its parents (*P* < 0.01). The analysis of reproductive traits revealed that 2nNCRC reaches sexual maturity at the age of one year, similarly to 2nCRC, and is significantly different from its parents, which reach sexual maturity at two years of age. The genotypic analysis showed that the chromosomal number, karyotype, and DNA content of 2nNCRC (Supplementary Tables S1 and S2; Supplementary Figs S2 and S3) are consistent with those of 2nCRC. Furthermore, the FISH results using 5S rDNA as the probe showed that 2nNCRC is more closely related to 2nCRC but is quite different from its parents (Fig. 2a–g), further demonstrating the similarity between 2nNCRC (F_1_–F_3_) and 2nCRC. The results of the 5S rDNA analyses also support the conclusion that 2nNCRC is very similar to 2nCRC. In summary, our results strongly indicate that 2nNCRC derived from the interspecific hybridization of 2nCOC (♀) × 2nBSB (♂) is phenotypically and genotypically similar to 2nCRC and its varieties (2nRCC and 2nJCC) that exist in nature. The artificial interspecific hybridization of female koi carp × male blunt snout bream repeatedly produced two types of new crucian carp-like homodiploid fish and one type of diploid gynogenesis carp, and the interspecific hybridization of female red crucian carp × male blunt snout bream formed tetraploid hybrids with a pair of barbels and a portion of the original genes of common carp^18–20^. All of the above-mentioned results suggest the existence of gene flow between *Cyprinus carpio* and *Carassius auratus* under natural conditions.

More than 32,500 fish species have been identified in nature^1^, and this number is higher than the total number of remaining vertebrate species. Our results indicate that distant hybridization resulting in homodiploid fish is an important homoploid speciation pathway and might play a key role in the formation of new species. Distant hybridization appears to facilitate speciation and adaptive radiation in both animals and plants^40^. Homodiploid hybrids can acquire novel combinations of phenotypes that allow them to colonize ecological niches that are inaccessible to both of the parental species^13^. Moreover, ecological divergence and selection for novel adaptations to new habitats might play important roles in promoting HHS^14^. In this study, 2nNCRC, a new homodiploid fish, exhibited marked genomic alterations that provide benefits for ecological adaptation and genetic evolution. This manuscript constitutes the first report of the formation of a homodiploid fish with phenotypic and genotypic variation through successive generations of hybridization. The establishment of the homodiploid fish line (F_1_–F_3_) provides an excellent model for investigating whether the phenotypic and genotypic changes can facilitate adaptation and promote biodiversity. Using this platform, we will be able to study the mechanisms of rapid homoploid speciation in distant hybridization. Our results reveal the occurrence of rapid homoploid speciation in the first generations of a distant hybridization and indicate the instability of the newly established homodiploid genome. Although homodiploid fish possess two sets of the 2nCOC-derived chromosomes, phenotypic and genotypic changes were clearly detected. The findings of this study provide novel insights into chromosomal evolution in vertebrates. In addition, the marked phenotypic and genotypic alterations are beneficial for genetic breeding because the homodiploid could provide an important source of gametes for the production of new allotriploid types with potential advantages of faster growth and improved disease resistance.

## Methods

### Ethics statement

All of the samples used in this study were cultured in ponds at the Protection Station of Polyploidy Fish, Hunan Normal University, and fed artificial feed. The guidelines established by the Administration of Affairs Concerning Animal Experimentation state that approval from the Science and Technology Bureau of China and the Department of Wildlife Administration is not necessary when the fish in question are neither rare nor near extinction (first- or second-class state protection level). Therefore, approval was not required for the experiments described in this manuscript. The fish were deeply anaesthetized with 100 mg/L MS-222 (Sigma-Aldrich, St. Louis, MO, USA) prior to dissection.

### Animals and crossing procedure

All of the natural material, specifically the common carp (2nCOC) and local crucian carp (2nCRC), were collected from Xiangjiang River in Hunan Province. The blunt-snout bream (2nBSB) were obtained from Liangzi Lake in Hubei Province. 2nCOC and 2nBSB were obtained from the Protection Station of Polyploidy Fish in Hunan Normal University. During the reproductive seasons (from April to June) in 2014–2016, 10 mature females and 10 mature males of 2nCOC and 2nBSB were selected as the maternal and paternal parents, respectively. The crosses were performed in two groups: in the first group, 2nCOC and 2nBSB were used as the maternal and paternal parents, respectively, whereas in the second group, the maternal and parental parents were switched. Mature eggs were fertilized, and the embryos were developed in culture dishes at a water temperature of 19–22 °C. In April 2015, the male and female 2nNCRC reached sexual maturity at the age of 1 year, similarly to 2nCRC, 2nRCC, and 2nJCC, and were mated to produce the second generation. By contrast, 2nGCOC, 2nGMCC, and 4nCB do not reach sexual maturity until the age of two to three years. From each cross, 2000 embryos were selected at random to determine the fertilization (number of embryos at the gastrula stage/number of eggs × 100%), hatching (number of hatched fry/number of eggs × 100%), and survival (number of adulthood/number of eggs × 100%) rates. Simultaneously, same-species matings of 2nCOC and 2nBSB were performed as controls. The hatched fry were then transferred to a pond for further culture.

The 2nCOC (♀) × 2nBSB (♂) cross resulted in four offspring: the new crucian carp-like homodiploid fish, a diploid natural gynogenesis common carp, a diploid gynogenesis scattered mirror carp (a variety of common carp), and a tetraploid hybrid. The reverse cross of 2nBSB (♀) × 2nCOC (♂) did not produce any living progeny, and the 2nNCRC self-cross resulted in a single offspring. Hereafter, the new crucian carp-like homodiploid fish produced by 2nCOC (♀) × 2nBSB (♂) is referred to as 2nNCRC, the self-cross offspring is denoted 2nNCRC-F_2_, and the subsequent self-cross offspring is called 2nNCRC-F_3_. The diploid natural gynogenesis common carp obtained from the 2nCOC (♀) × 2nBSB (♂) cross was denoted 2nGCOC, the diploid scattered mirror carp (a variety of common carp) derived from 2nCOC (♀) × 2nBSB (♂) was abbreviated 2nGMCC, and the tetraploid hybrid produced by 2nCOC (♀) × 2nBSB (♂) was denoted 4nCB.

The 2nCOC (♀) × 2nBSB (♂) cross showed high fertilization (85.8%) and hatching (72.1%) rates, but the offspring presented a relatively low survival rate (38.9%). The same-species mating of 2nCOC resulted in fertilization, hatching, and survival rates equal to 90.6, 86.3, and 78.9%, respectively, and that of 2nBSB showed rates of 92.9, 88.2, and 73.4%, respectively. In addition, the fertilization, hatching, and survival rates found for the 2nNCRC self-cross offspring were 90.2, 85.6, and 75.1%, respectively. No living progeny were obtained from the reverse cross of 2nBSB (♀) × 2nCOC (♂). The ratio statistics of the hybrid offspring in 2014–2016 are shown in Table 1.

### Preparation of chromosome spreads and measurement of DNA content

For the determination of ploidy, kidney tissues from 10 2nNCRC, 2nNCRC-F_2_, 2nGCOC, 2nGMCC, and 4nCB at one year of age (50 fish in total) were used for chromosome counts. The preparations were made according to the method described by Liu *et al*.^4^ with minor modifications. After culturing for two to three days at 20–22 °C, the samples were injected one to three times with concanavalin at a dose of 6–15 µg/g body weight at an interval of 12–24 hours. Two to three hours prior to dissection, each sample was injected with colchicine at a dose of 4–6 µg/g body weight. The kidney tissue was ground in 0.9% NaCl, subjected to hypotonic treatment with 0.075 M KCl at 37 °C for 40–60 min, and then fixed three times in 3:1 methanol-acetic acid. The cells were added dropwise to cold, wet slides and stained with 4% Giemsa for 40–60 min. The chromosome shape and numbers were analysed under a light microscope. We were analysed 200 metaphase spreads (20 metaphase spreads in each sample) for each type of fish. The preparations were examined under an oil lens at a magnification of 330×.

The DNA contents of erythrocytes from the 2nCOC, 2nBSB, 2nCOC (♀) × 2nBSB (♂) offspring and the self-cross 2nNCRC offspring were measured using a flow cytometer (Cell Counter Analyser, Partec, Germany). We collected 1–2 ml of blood from the caudal vein of each individual using a syringe containing ~200–300 units of sodium heparin. The blood samples were processed following the method described Liu *et al*.^4^ and investigated under the same conditions. The 2nCOC and 2nBSB DNA contents were used as controls. For comparison of the DNA contents of the offspring with those of either 2nCOC or 2nBSB, we used the χ^2^ test with a Yates correction to test the deviation from the expected values.

### Morphological traits

Several countable and measurable traits were assessed in 20 one-year-old 2nCOC, 2nBSB, 2nNCRC, 2nGCOC, 2nGMCC, 4nCB, 2nCRC, 2nRCC, and 2nJCC individuals (total = 180). The countable traits include the numbers of dorsal, abdominal, and anal fins and the numbers of lateral, upper lateral, and lower lateral scales. The measurable traits include the average values of the whole length (WL), body length (BL) and width (BW), head length (HL) and width (HW), and tail length (TL) and width (TW). Additionally, the average ratios of the body length to whole length (BL/WL), body width to body length (BW/BL), head length to body length (HL/BL), head width to head length (HW/HL), tail width to tail length (TW/TL), and head width to body width (HW/BW) were also calculated.

SPSS Statistics 17.0 (IBM Corp., NY, USA) was used to analyse the covariance in the morphological traits among the four types of offspring and their parents.

### Fluorescence *in situ* hybridization and microscopy

The *5S* gene probe hybridized to the metaphase 2nNCRC, 2nNCRC-F_2_, 2nGCOC, 2nGMCC, and 4nCB chromosomes. The fluorescence *in situ* hybridization (FISH) *5S* gene and species-specific centromere probes were constructed for 2nCRC and 2nRCC and amplified by PCR using the *5S* P1 and *5S* P2R primers (5′-GCTATGCCCGATCTCGTCTGA-3′ and 5′-CAGGTTGGTATGGCCGTAAGC-3′, respectively)^20^. The FISH probes were produced by Dig-11-dUTP labelling (using a nick translation kit, Roche, Germany) of the purified PCR products. FISH was performed according to the method described by He *et al*.^33^. For each type of fish, 200 metaphase spreads (20 for each sample) of the chromosomes were analysed. The *5S* gene probe (GenBank Accession No. GQ485556) hybridized to metaphase 2nCOC, 2nBSB, 2nNCRC, 2nNCRC-F_2_, 2nGCOC, 2nGMCC, and 4nCB chromosomes (Fig. 2a–g).

### Gonadal structure and spermatozoa phenotype

One female 2nNCRC individual was randomly sampled (only six living 2nNCRC-F_1_ of 2nCOC (♀) × 2nBSB (♂) were observed in 2014; four females and two males). The 2nNCRC gonad was fixed in Bouin’s solution for the preparation of tissue sections. Paraffin-embedded sections were cut and stained with haematoxylin and eosin. The gonadal structure was observed under a light microscope and photographed with a Pixera Pro 600ES (Pixera Corporation, Santa Clara, CA, USA).

2nNCRC, 2nGCOC, and 2nGMCC semen samples were collected with a clean sucker and transferred into a 2.5% glutaraldehyde solution. The samples were then centrifuged at 2000 × *g* for 1 minute and fixed in a 4% glutaraldehyde solution overnight and in a 1% osmic acid solution for 2 hours. The spermatozoa were dehydrated in alcohol, added dropwise onto slides, desiccated, subjected to atomized gilding and analysed with an X-650 (Hitachi) SEM scanning electron microscope (Nikon, Japan).

### Genomic DNA extraction, PCR, and sequencing

The total genomic DNA from the peripheral blood cells of 2nCOC, 2nBSB, and their offspring was extracted using a phenol/chloroform extraction method as described by Sambrook *et al*.^41^. A set of primers (*5S* P1, 5′-GCTATGCCCGATCTCGTCTGA-3′; *5S* P2R, 5′-CAGGTTGGTATGGCCGTAAGC-3′) was designed and synthesized to amplify the *5S* rRNA genes and their non-transcribed spacer regions directly from genomic DNA according to Qin *et al*.^34^. The PCR reactions were performed in a volume of 25 µL with approximately 20 ng of genomic DNA, 1.5 mM MgCl_2_, 250 µM of each dNTP, 0.4 µM of each primer, and 1.25 U of Taq polymerase (TaKaRa, Dalian, China). The thermal programme consisted of an initial denaturation step at 94 °C for 5 min followed by 30 cycles of 94 °C for 35 s, 59 °C for 35 s, and 72 °C for 35 s and a final extension step at 72 °C for 5 min. The amplification products were separated on a 1.5% agarose gel using TBE buffer. After electrophoresis, the DNA fragments were purified using a gel extraction kit (Sangon, Shanghai, China) and ligated to the pMD18-T vector. The plasmids were transformed into *Escherichia coli DH5α*, and the DNA fragments inserted into the pMD18-T vector were sequenced using an automated DNA sequencer (ABI PRISM 3730, Applied Biosystems, CA, USA). The sequence homology and variation among the fragments amplified from 2nCOC, 2nBSB, 2nNCRC, 2nGCOC, 2nGMCC, and 4nCB were analysed using BioEdit^42^ and Clustal W^43^.

### PCR polymorphism

The PCR amplification of the DNA from 2nCOC, 2nBSB, and their offspring with the *5S* primers P_1_ and P_2_R produced distinctive band patterns: two bands in 2nCOC (~200 and 400 bp), two bands in 2nBSB (~200 and 400 bp), four bands in 2nNCRC (~200, 350, 400, and 500 bp), two bands in 2nGCOC (~200 and 400 bp), two bands in 2nGMCC (~200 and 400 bp), and four bands in 4nCB (~200, 350, 400, and 500 bp; Supplementary Fig. S4).

In total, 390 clones were sequenced to examine the different 5S rDNA patterns: 20 2nCOC, 30 2nBSB, 120 2nNCRC, 60 2nGCOC, 60 2nGMCC, and 100 4nCB clones (Supplementary Table S3). PCR revealed two fragments in 2nCOC (203 and 406 bp), three in 2nBSB (188, 374, and 376 bp), 12 in 2nNCRC (196, 205, 339, 340, 341, 374, 398, 406, 410, 478, 480, and 493 bp), three in 2nGCOC (203, 205, and 406 bp), three in 2nGMCC (203, 205, and 406 bp), and 10 in 4nCB (196, 203, 204, 205, 340, 386, 406, 480, 493, and 507 bp) (Supplementary Table S3). A sequence analysis showed that 2nNCRC, 2nGCOC, 2nGMCC, and 4nCB possess either two or four similarly sized *5S* PCR products of either 196, 203, or 205 bp, which are not distinguishable on the agarose gel, where they appear as a single ~200-bp band. The sequence analysis also revealed three similarly sized *5S* PCR products of 339, 340, and 341 bp in 2nNCRC that were not distinguishable on the agarose gel, where they appear as a single ~350-bp band. Similarly, 2nBSB, 2nNCRC, and 4nCB possess either two or four similarly sized *5S* PCR products of 374, 376, 386, 398, 406, or 410 bp that were not distinguishable on the agarose gel, appearing as a single ~400-bp band. 2nNCRC and 4nCB possess three similarly sized *5S* PCR products of 478, 480, 493, or 507 bp that were not distinguishable on the agarose gel, where they appear as a single ~500-bp band.

## Electronic supplementary material

Supplementary information.
