# Supplementary material for: A new type of homodiploid fish derived from the interspecific hybridization of female common carp × male blunt snout bream
Source: Sci Rep. 2017 Jun 23;7:4189. doi: 10.1038/s41598-017-04582-z (PMC5482800; doi:10.1038/s41598-017-04582-z)
Supplement: Supplementary file 1 — Supplementary information. [file 41598_2017_4582_MOESM1_ESM.pdf]

Supplementary Information Appendix for:

A new type of homodiploid fish derived from the interspecific hybridization of female common carp × male blunt snout bream

Shi Wang[1,2,*], Xiaolan Ye[1,2,*], Yude Wang[1,2,*], Yuting Chen[1,2], Bowen Lin[1,2], Zhenfeng Yi[1,2], Zhuangwen Mao[1,2], Fangzhou Hu[1,2], Rurong Zhao[1,2], Juan Wang[1,2], Rong Zhou[1,2], Li Ren[1,2], Zhanzhou Yao[1,2], Min Tao[1,2], Chun Zhang[1,2], Jun Xiao[1,2], Qinbo Qin[1,2], Shaojun Liu[1,2,†]

[1] State Key Laboratory of Developmental Biology of Freshwater Fish, Hunan Normal University, Changsha, 410081, Hunan, P.R. China

[2] College of Life Sciences, Hunan Normal University, Changsha, 410081, Hunan, P.R. China

[†]Corresponding author: Professor Shaojun Liu, E-mail: lsj@hunnu.edu.cn

[*]These authors contributed equally to this work.

Figure Legends

Supplementary Figure S1: Crossing procedure and formation of the different ploidy offspring. The chromosomes of common carp (2nCOC) and blunt snout bream (2nBSB) are marked by the blue and red color, respectively. In the first generation of common carp (*Cyprinus carpio*, 2nCOC ♀, *Cyprininae*, 2n=100) × blunt snout bream (*Megalobrama amblycephala*, 2nBSB ♂, *Cultrinae*, 2n=48), the new crucian carp-like homodiploid fish (2n=100, 2nNCRC), diploid gynogenetic common carp (2n=100, 2nGCOC), diploid gynogenetic mirror common carp (a variety of common carp; 2n=100, 2nGMCC), and tetraploid hybrids (4n=148, 4nCB) are produced. The new crucian carp-like homodiploid fish of F_2 (2n=100, 2nNCRC-F_2) is produced in the second generation of 2nNCRC by self-crossing. The new crucian carp-like homodiploid fish of F_3 (2n=100, 2nNCRC-F_3) is produced in the third generation of 2nNCRC-F_2 by self-crossing.

Supplementary Figure S2: Cytometric histograms of DNA fluorescence for 2nBSB, 2nCOC, 2nGCOC, 2nGMCC, 2nNCRC, 4nCB, and 2nNCRC-F_2. The mean DNA content of 2nBSB is 65.98. The mean DNA content of 2nCOC is 101.72. The mean DNA content of 2nGCOC is 104.28, which is equal to that of 2nCOC (P>0.01), suggesting that it has two sets of 2nCOC-derived chromosomes (2n). The mean DNA content of 2nGMCC is 98.36, which is equal to that of 2nCOC (P>0.01), suggesting that it has two sets of 2nCOC-derived chromosomes (2n). The mean DNA content of 2nNCRC is 99.17, which is equal to that of 2nCOC ($P>0.01$), suggesting that it has two sets of 2nCOC-derived chromosomes (2n). The mean DNA content of

4nCB is 162.64, which is equal to the sum of that of 2nCOC and 2nBSB (P>0.01), suggesting that it has two sets of 2nCOC-derived chromosomes and two sets of 2nBSB-derived chromosomes (4n). The mean DNA content of 2nNCRC-F_2 is 94.26, which is equal to that of 2nCOC content (P>0.01), suggesting that 2nNCRC-F_2 has two sets of 2nCOC-derived chromosomes (2n).

Supplementary Figure S3: Chromosome spreads at metaphase, and corresponding karyotypes of 2nCOC, 2nBSB and their offspring. (a) The 100 chromosomes of 2nCOC, with no large submetacentric chromosome. **(b)** The 48 chromosomes of 2nBSB, with a pair of the largest submetacentric chromosomes indicated (solid arrows). **(c)** The 100 chromosomes of 2nNCRC, with no large submetacentric chromosome. **(d)** The karyotype of 2nNCRC is 22m+34sm+22st+22t, in which no large submetacentric chromosome is detected, comprising two sets of chromosomes from 2nCOC. **(e)** The 100 chromosomes of 2nNCRC-F_2, with no large submetacentric chromosome. **(f)** The karyotype of 2nNCRC-F_2 is 22m+34sm+22st+22t, in which no large submetacentric chromosome is detected, comprising two sets of chromosomes from 2nCOC. **(g)** The 100 chromosomes of 2nGCOC, with no large submetacentric chromosome. **(h)** The karyotype of 2nGCOC is 22m+34sm+22st+22t, in which no large submetacentric chromosome is detected, comprising two sets of chromosomes from 2nCOC. **(i)** The 100 chromosomes of 2nGMCC, with no large submetacentric chromosome. **(j)** The karyotype of 2nGMCC is 22m+34sm+22st+22t, in which no large submetacentric chromosome is detected, comprising two sets of chromosomes from 2nCOC. **(k)** The 148 chromosomes of

4nCB, with a pair of the largest submetacentric chromosomes indicate (solid arrows). **(l)** The karyotype of 4nCB is 40m+56sm+30st+22t, consisting of two sets of chromosomes from 2nCOC and two sets from 2nBSB. The solid arrow indicates a pair of the largest submetacentric chromosomes similar to those of 2nBSB. Scale bars in a–l, 3 μm.

Supplementary Figure S4: DNA bands amplified from 2nCOC, 2nBSB and their offspring. M: DNA ladder markers (200bp increments); **lane 1:** two DNA bands (~200 and 400bp) from 2nCOC; **lane 2:** two DNA bands (~200 and 400bp) from 2nBSB; **lane 3:** two DNA bands (~200 and 400bp) from 2nGCOC; **lane 4:** two DNA bands (~200 and 400bp) from 2nGMCC; **lane 5:** four DNA bands (~200, 350, 400 and 500bp) from 2nNCRC; **lane 6:** four DNA bands (~200, 350, 400 and 500bp) from 4nCB.

SI Appendix

I. SI Figures

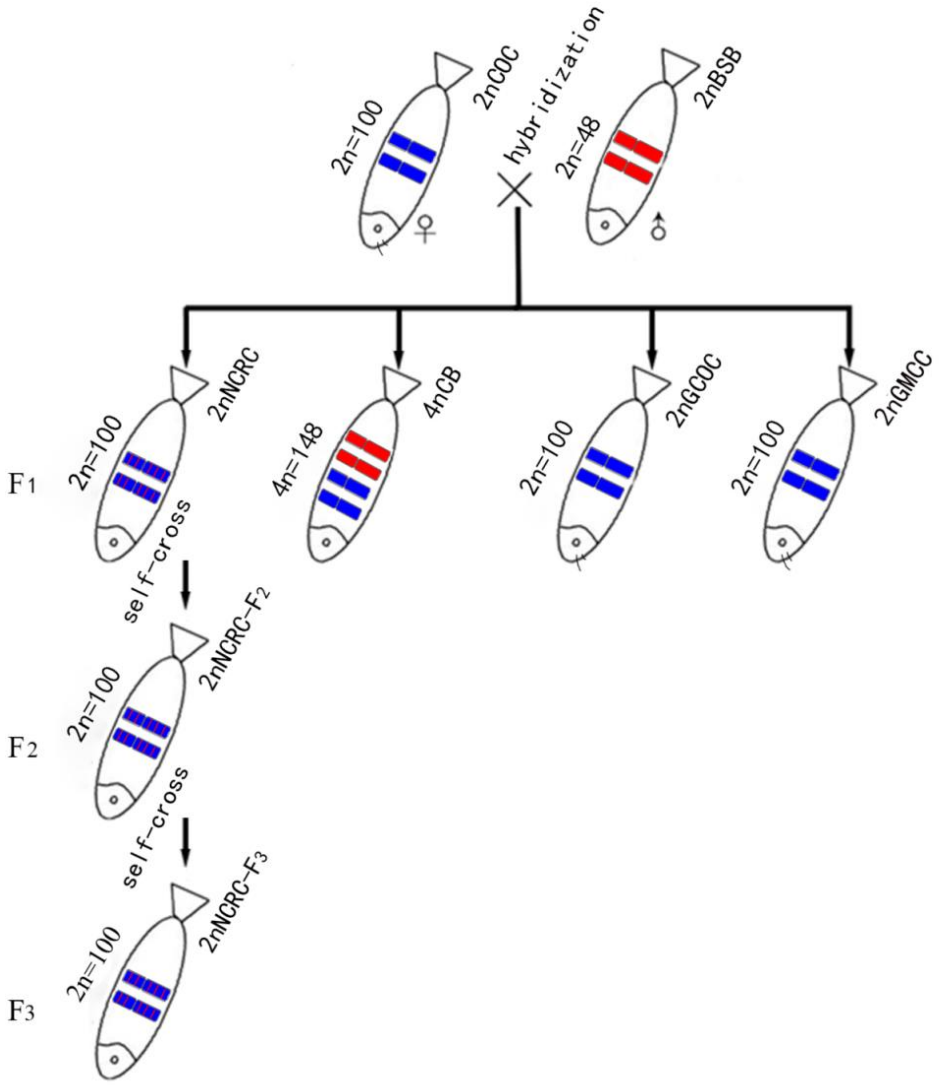

Supplementary Figure S1: Crossing procedure and formation of the different ploidy offspring. The chromosomes of common carp (2nCOC) and blunt snout bream (2nBSB) are marked by the blue and red color, respectively. In the first generation of common carp (*Cyprinus carpio*, 2nCOC ♀, *Cyprininae*, 2n=100) ×blunt snout bream (*Megalobrama amblycephala*, 2nBSB ♂, *Cultrinae*, 2n=48), the new crucian carp-like homodiploid fish (2n=100, 2nNCRC), diploid gynogenetic common carp (2n=100, 2nGCOC), diploid gynogenetic mirror common carp (a variety of common carp; 2n=100, 2nGMCC), and tetraploid hybrids (4n=148, 4nCB)are produced. The new crucian carp-like homodiploid fish of F_2 (2n=100, 2nNCRC-F_2) is produced in the second generation of 2nNCRC by self-crossing. The new crucian carp-like homodiploid fish of F_3 (2n=100, 2nNCRC-F_3) is produced in the third generation of 2nNCRC-F_2 by self-crossing.

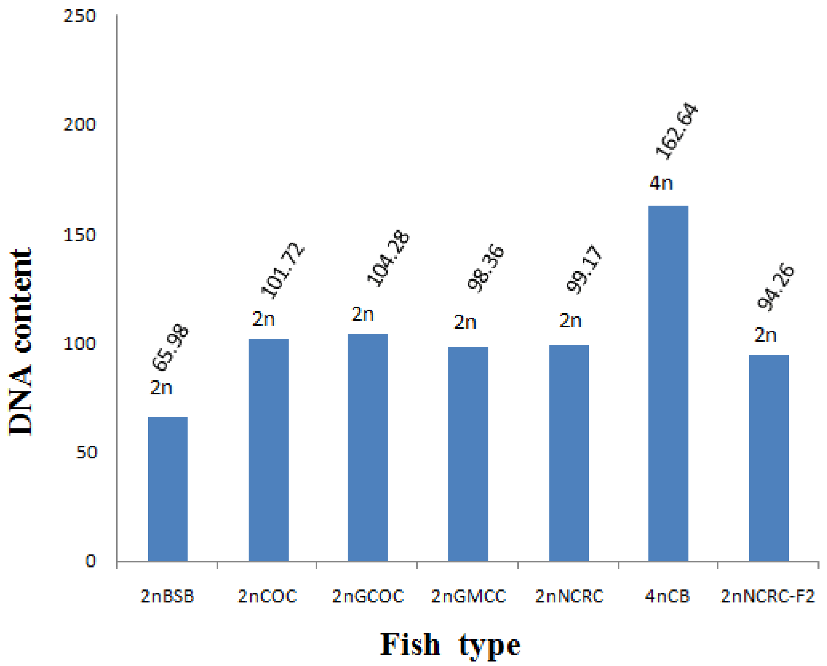

Supplementary Figure S2: Cytometric histograms of DNA fluorescence for 2nBSB, 2nCOC, 2nGCOC, 2nGMCC, 2nNCRC, 4nCB, and 2nNCRC-F_2. The mean DNA content of 2nBSB is 65.98. The mean DNA content of 2nCOC is 101.72. The mean DNA content of 2nGCOC is 104.28, which is equal to that of 2nCOC (P>0.01), suggesting that it has two sets of 2nCOC-derived chromosomes (2n). The mean DNA content of 2nGMCC is 98.36, which is equal to that of 2nCOC (P>0.01), suggesting that it has two sets of 2nCOC-derived chromosomes (2n). The mean DNA content of 2nNCRC is 99.17, which is equal to that of 2nCOC ($P>0.01$), suggesting that it has two sets of 2nCOC-derived chromosomes (2n). The mean DNA content of 4nCB is 162.64, which is equal to the sum of that of 2nCOC and 2nBSB (P>0.01), suggesting that it has two sets of 2nCOC-derived chromosomes and two sets of 2nBSB-derived chromosomes (4n). The mean DNA content of 2nNCRC-F_2 is 94.26, which is equal to that of 2nCOC content (P>0.01), suggesting that 2nNCRC-F_2 has two sets of 2nCOC-derived chromosomes (2n).

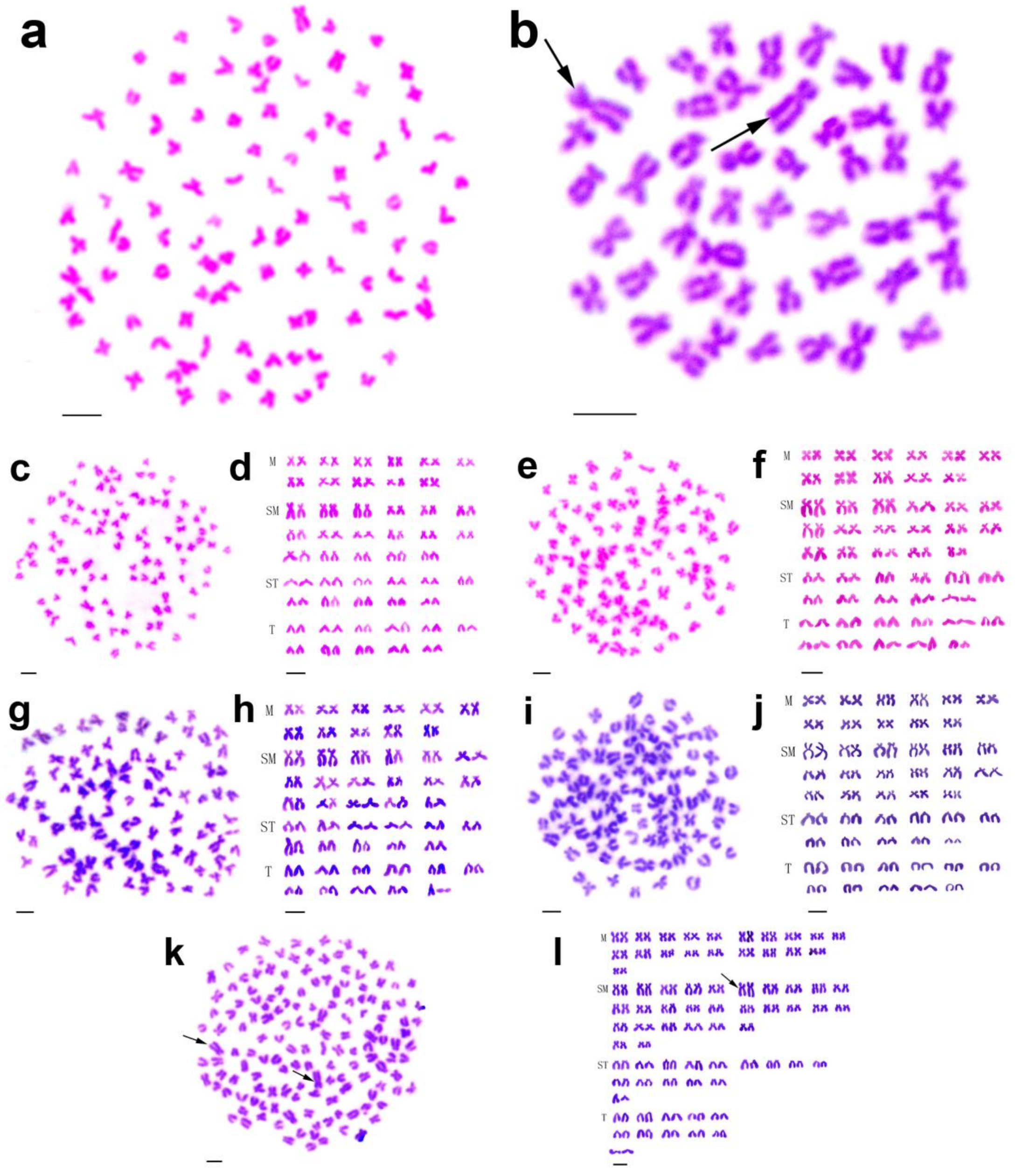

Supplementary Figure S3: Chromosome spreads at metaphase, and corresponding karyotypes of 2nCOC, 2nBSB and their offspring. (a) The 100 chromosomes of 2nCOC, with no large submetacentric chromosome. **(b)** The 48 chromosomes of 2nBSB, with a pair of the largest submetacentric chromosomes indicated (solid arrows). **(c)** The 100 chromosomes of 2nNCRC, with no large submetacentric chromosome. **(d)** The karyotype of 2nNCRC is 22m+34sm+22st+22t, in which no large submetacentric chromosome is detected, comprising two sets of chromosomes from 2nCOC. **(e)** The 100 chromosomes of 2nNCRC-F_2, with no large submetacentric chromosome. **(f)** The karyotype of 2nNCRC-F_2 is 22m+34sm+22st+22t, in which no large submetacentric chromosome is detected, comprising two sets of chromosomes from 2nCOC. **(g)** The 100 chromosomes of 2nGCOC, with no large submetacentric chromosome. **(h)** The karyotype of 2nGCOC is 22m+34sm+22st+22t, in which no large submetacentric chromosome is detected,

comprising two sets of chromosomes from 2nCOC. **(i)** The 100 chromosomes of 2nGMCC, with no large submetacentric chromosome. **(j)** The karyotype of 2nGMCC is 22m+34sm+22st+22t, in which no large submetacentric chromosome is detected, comprising two sets of chromosomes from 2nCOC. **(k)** The 148 chromosomes of 4nCB, with a pair of the largest submetacentric chromosomes indicate (solid arrows). **(l)** The karyotype of 4nCB is 40m+56sm+30st+22t, consisting of two sets of chromosomes from 2nCOC and two sets from 2nBSB. The solid arrow indicates a pair of the largest submetacentric chromosomes similar to those of 2nBSB. Scale bars in a–l, 3 μm.

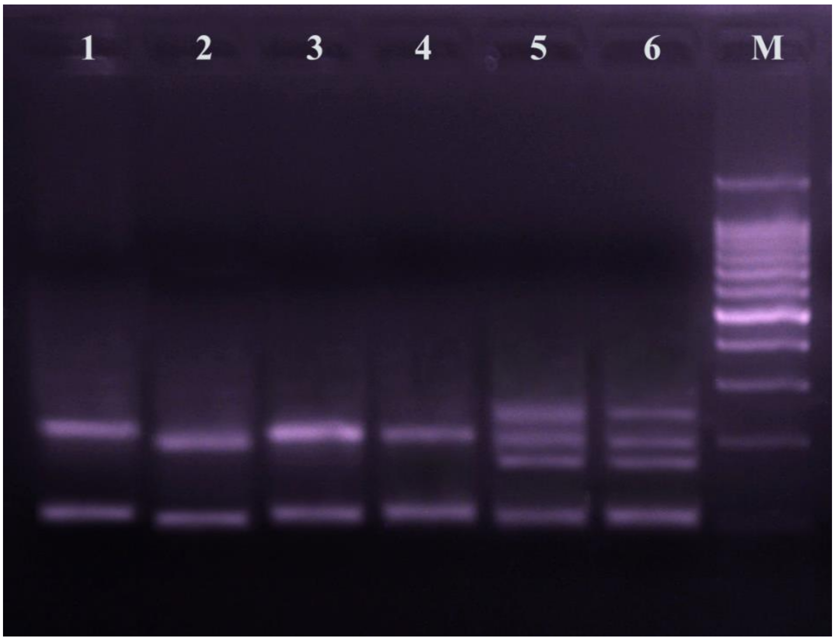

Supplementary Figure S4: DNA bands amplified from 2nCOC, 2nBSB and their offspring. M: DNA ladder markers (200bp increments); **lane 1:** two DNA bands (~200 and 400bp) from 2nCOC; **lane 2:** two DNA bands (~200 and 400bp) from 2nBSB; **lane 3:** two DNA bands (~200 and 400bp) from 2nGCOC; **lane 4:** two DNA bands (~200 and 400bp) from 2nGMCC; **lane 5:** four DNA bands (~200, 350, 400 and 500bp) from 2nNCRC; **lane 6:** four DNA bands (~200, 350, 400 and 500bp) from 4nCB.

II. SI Tables

Supplementary Table S1: The mean DNA content in 2nBSB, 2nCOC, 2nNCRC, 2nNCRC-F_2, 2nGCOC, 2nGMCC, and 4nCB

Fish type	Mean [a] DNA content	Ratio	
		Observed	Expected
2nBSB	65.98		
2nCOC	101.72		
2nNCRC	99.17	2nNCRC/2nCOC=0.97[b]	1
2nNCRC-F_2	94.26	2nNCRC-F_2/2nCOC=0.93[b]	1
2nGCOC	104.28	2nGCOC/2nCOC=1.03[b]	1
2nGMCC	98.36	2nGMCC/2nCOC=0.97[b]	1
4nCB	162.64	4nCB/(2nCOC+2nBSB)=0.97[b]	1

[a] The intensity of fluorescence (unit, channel).

[b]The observed ratio is not significantly different (P>0.05) from the expected ratio.

Supplementary Table S2: The chromosome number in 2nCOC, 2nBSB, 2nNCRC, 2nNCRC-F$_2$, 2nGCOC, 2nGMCC, and 4nCB offspring

Fish type	Distribution of chromosome number						
	No. in metaphase	<48[a]	48	<100[a]	100	<148[a]	148
2nCOC	200			9	191		
2nBSB	200	13	187				
2nNCRC	200			16	184		
2nNCRC-F$_2$	200			17	183		
2nGCOC	200			12	188		
2nGMCC	200			15	185		
4nCB	200					38	162

[a]The chromosome number is less than what they should be, owning to the loss of chromosomes in the procedure of chromosome preparation.

Supplementary Table S3: The results of 5S rDNA sequences

Samples	Number of sequenced clones	PCR bands			
		~200bp[a]	~350bp[a]	~400bp[a]	~500bp[a]
**2nCOC**	20	10 clones of 203bp (class I)	Absent	10 clones of 406bp (class I)	Absent
**2nBSB**	30	10 clones of 188bp (class II)	Absent	8 clones of 374bp (class II-V_1); 12 clones of 376bp (class II)	Absent
**2nNCRC**	120	5 clones of 196bp (class I-V_1); 15 clones of 205bp (class I-V_1)	9 clones of 339bp (class I-V_2); 13 clones of 340bp (class I-V_2); 8 clones of 341bp (class I-V_2)	14 clones of 374bp (class II-V_1); 7 clones of 398bp (class II-V_1); 12 clones of 406bp (class II-V_1); 7 clones of 410bp (class II-V_1)	12 clones of 478bp (class I-V_3); 8 clones of 480bp (class I-V_3); 10 clones of 493bp (class I-V_3)
**2nGMCC**	60	24 clones of 203bp (class I); 16 clones of 205bp (class I)	Absent	20 clones of 406bp (class I)	Absent
**2nGCOC**	60	26 clones of 203bp (class I) ; 14 clones of 204bp (class I)	Absent	20 clones of 406bp (class I)	Absent
**4nCB**	100	6 clones of 196bp (class I-V_1); 15 clones of 203bp (class I); 8 clones of 204bp (class I); 11 clones of 205bp (class I-V_1)	10 clones of 340bp (class I-V_2)	8 clones of 386bp (class II-V_1); 12 clones of 406bp (class II-V_1)	7 clones of 480bp (class I-V_3); 14 clones of 493bp (class I-V_3); 9 clones of 507bp (class I-V_3)

[a]The approximate size of PCR bands on the agarose gel.
